# The multi-faceted functioning portrait of LRF/*ZBTB7A*

**DOI:** 10.1186/s40246-019-0252-0

**Published:** 2019-12-10

**Authors:** Caterina Constantinou, Magda Spella, Vasiliki Chondrou, George P. Patrinos, Adamantia Papachatzopoulou, Argyro Sgourou

**Affiliations:** 1 Biology laboratory, School of Science and Technology, Hellenic Open University, Patras, Greece; 2 Laboratory of Pharmacology, Department of Medicine, University of Patras, Patras, Greece; 3 Laboratory for Molecular Respiratory Carcinogenesis, Department of Physiology, Medical Faculty, University of Patras, Patras, Greece; 4 Laboratory of Pharmacogenomics and Individualized Therapy, Department of Pharmacy, School of Health Sciences, University of Patras, Patras, Greece; 5 Department of Pathology, College of Medicine and Health Sciences, United Arab Emirates University, Al-Ain, UAE; 6 Zayed Center of Health Sciences, United Arab Emirates University, Al-Ain, UAE; 7 Laboratory of General Biology, Medical Faculty, University of Patras, Patras, Greece

**Keywords:** Zinc finger and BTB domain transcription factor, Hematopoietic stem cell differentiation, Oncogene, Tumor-suppressor gene, Apoptosis, Glycolysis, Adipogenesis, Thymic insulin expression

## Abstract

Transcription factors (TFs) consisting of zinc fingers combined with BTB (for broad-complex, tram-track, and bric-a-brac) domain (ZBTB) are a highly conserved protein family that comprises a multifunctional and heterogeneous group of TFs, mainly modulating cell developmental events and cell fate. LRF/*ZBTB7A*, in particular, is reported to be implicated in a wide variety of physiological and cancer-related cell events. These physiological processes include regulation of erythrocyte maturation, B/T cell differentiation, adipogenesis, and thymic insulin expression affecting consequently insulin self-tolerance. In cancer, LRF/*ZBTB7A* has been reported to act either as oncogenic or as oncosuppressive factor by affecting specific cell processes (proliferation, apoptosis, invasion, migration, metastasis, etc) in opposed ways, depending on cancer type and molecular interactions. The molecular mechanisms via which LRF/*ZBTB7A* is known to exert either physiological or cancer-related cellular effects include chromatin organization and remodeling, regulation of the Notch signaling axis, cellular response to DNA damage stimulus, epigenetic-dependent regulation of transcription, regulation of the expression and activity of NF-κB and p53, and regulation of aerobic glycolysis and oxidative phosphorylation (Warburg effect). It is a pleiotropic TF, and thus, alterations to its expression status become detrimental for cell survival. This review summarizes its implication in different cellular activities and the commonly invoked molecular mechanisms triggered by LRF/*ZBTB7A*’s orchestrated action.

## Introduction

Zinc finger and BTB domain containing 7A (*ZBTB7A*) appears in the literature with several synonyms: POK erythroid myeloid ontogenic factor or POZ and Krüppel-erythroid myeloid ontogenic factor (Pokemon), factor binding IST protein-1 (FBI-1), HIV-1 inducer of short transcripts binding protein, TTF-I-interacting peptide 21, DKFZp547O146, etc. The lymphoma/leukemia-related factor (LRF) preferentially refers to the protein product encoded by the *ZBTB7A* gene.

Characteristic domains of the LRF/*ZBTB7A* transcription factor are the four C-terminal Krüppel-type zinc fingers with a sequence-specific DNA-binding capacity and the N-terminal (broad-complex, tram-track, and bric-a-brac) BTB domain, capable for the formation of homo- or hetero-dimers. These features provide LRF/*ZBTB7A* with collaboration abilities and highly contextual activities towards cellular function, such as transcription co-repressor activity, protein and histone acetyltransferase binding, proximal promoter sequence-specific DNA-binding, and DNA-binding with consecutive attraction and modulation of other TFs activities.

LRF/*ZBTB7A* exerts its action within the nucleus compartment; herein, cell responses may differ in different tissues and local cell microenvironments. Cell processes disturbed by the aberrant expression of LRF/*ZBTB7*A TF are discussed in the present manuscript in an effort to give prominence to its both widespread gene regulatory effects and components of cellular life-cycle affected. Its basic ability to cooperate and attract various complexes to the broader “targeted” regulated genome area determines not only its pleiotropic and sometimes conflicting action, but also its potential to act on a case-by-case basis, depending on the epigenetic profile of the genome, due to its preferred binding sites within rich CG regions [1–3]. In humans, it has been extensively implicated in cell fate and cell differentiation abnormalities, highly significant for malignant manifestations and disease outcome. Besides its potent implications to the hematopoietic tissue, lymphoid development, and adipogenesis, involvement in specific downstream intracellular pathways, cell cycle, apoptosis, and glycolysis is presented, along with a fine tuning on the insulin’s thymic expression regulation, highlighting the universal role of this TF towards genome regulation.

### The LRF/*ZBTB7A* repertoire during erythroid development

**Fig. 1:**
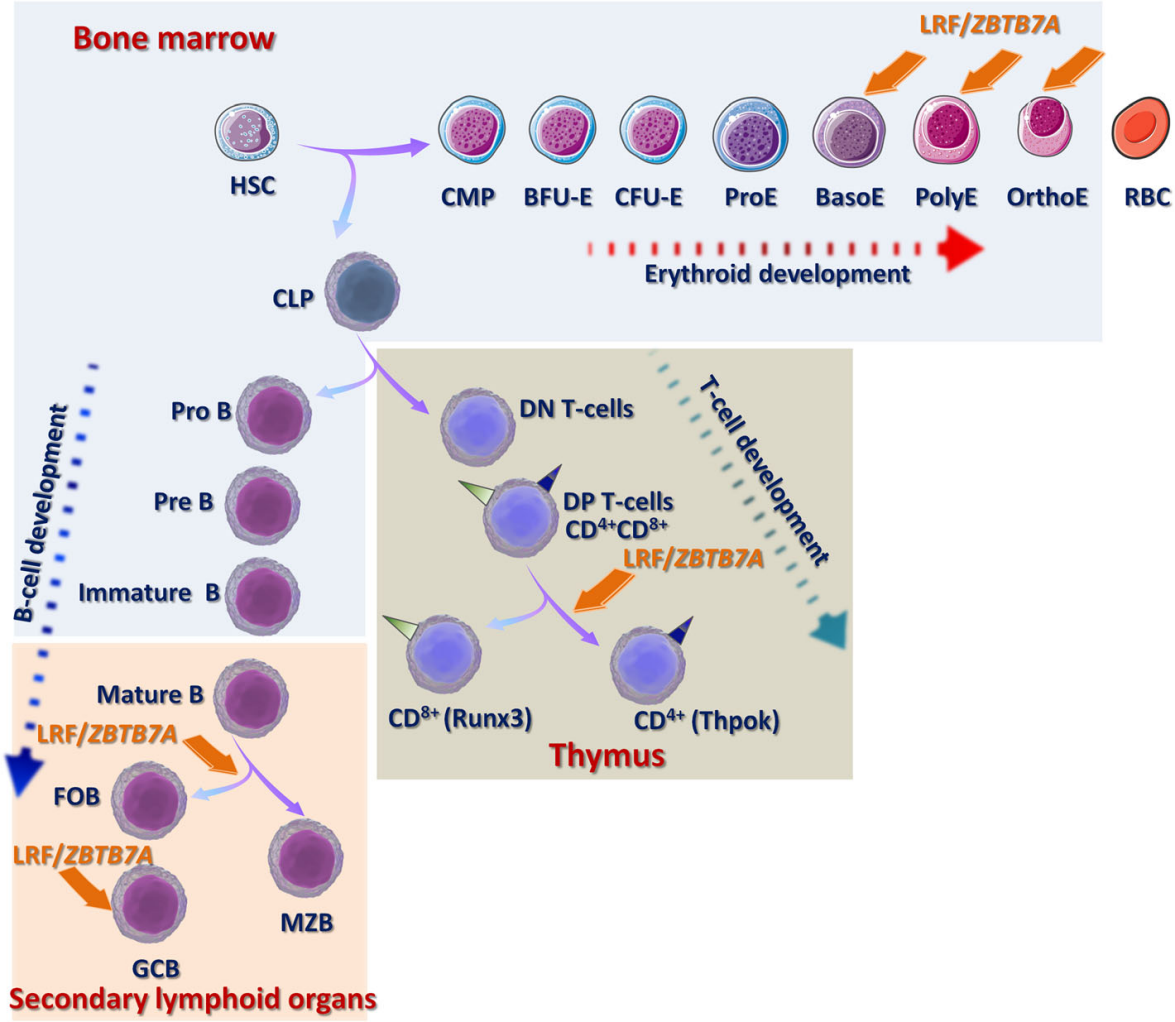
LRF/*ZBTB7A* regulates hematopoiesis and lymphoid development. LRF/*ZBTB7A* contribution in specific stages of cell differentiation is indicated. Abbreviations: HSC, hematopoietic stem cell; CMP, common myeloid progenitors; BFU-E, burst-forming unit-erythroid; CFU-E, colony-forming unit-erythroid; ProE, proerythroblasts; BasoE, basophilic erythroblasts; PolyE, polychromatophilic erythroblasts; OrthoE, orthochromatic erythroblasts; RBC, matured erythrocytes; CLP, common lymphoid progenitors; ProB, progenitor B cell; PreB, precursor B cell; DN T cell, double negative or CD4^-^/8^-^ negative; DP T cell, double positive or CD4^+^/8^+^ positive; CD4^+^ single positive expressing Thpok (T-helper inducing POZ/Krüppel-like factor); CD8^+^ single positive expressing the Runx3; FOB, follicular B cells; MZB, marginal zone B cells; GCΒ, germinal center B cells

Hematopoietic stem cells (HSCs) residing in specified compartments of the bone marrow, called “niche,” coordinate the replenishment of all types of blood cells through a series of lineage restriction steps, leading to pluripotency depletion and increasing commitment to a single pathway (lineage commitment). Differentiation of HSCs to mature erythrocytes involves the transition to common myeloid progenitors (CMP), early erythroid progenitors (burst-forming unit-erythroid, BFU-E), late erythroid progenitors (colony-forming unit-erythroid, CFU-E), and the morphologically recognizable erythroid precursors (proerythroblasts, basophilic erythroblasts, polychromatophilic erythroblasts, and orthochromatic erythroblasts) that undergo terminal maturation to erythrocytes by extruding their nuclei and progressively losing cytoplasmic organelles [4]. During effective erythropoiesis LRF/*ZBTB7A,* activated by the erythroid-specific transcription factor GATA1, directly binds to the promoter of the pro-apoptotic factor BCL2 like 11 (*BIM*), inhibits its transcription and suppresses cell apoptosis, ensuring the final production of erythroid cells [5]. Furthermore, KLF1 (Krüpel-like factor 1) cooperates with GATA1 to upregulate LRF/*ZBTB7A* expression in both human and mouse erythroid cell lines [6]. By its turn, LRF/*ZBTB7A* binds at GATA1 occupancy sites of a number of direct GATA1 target genes and recruits the chromatin repressive complex Polycomb Repressive Complex 2 (PRC2), directing epigenetic gene silencing [7] (Fig. 1).

Masuda and coworkers [8] demonstrated that LRF/*ZBTB7A* depletion reactivates the embryonic/fetal hemoglobin expression in adult mice and the γ-globin expression in human erythroblasts, due to permissive changes at the local chromatin conformation surrounding the γ-globin genes. LRF/*ZBTB7A* acts as a γ-globin repressor, during hemoglobin transversion from the fetal (HbF) to adult (HbA), independently from the master transcription factor B cell lymphoma/leukemia 11A (*BCL11A*), via the recruitment of the Nucleosome Remodeling and Deacetylase (NuRD) chromatin remodeling complex. In support to this mechanism, *Zbtb7a*^-^/^-^ mice embryos exhibit early lethal anemia, while conditional inactivation of Lrf/*Zbtb7a* in adult mice blocks the terminal erythroid differentiation and leads to macrocytic anemia [5].

Reactivation of HbF expression, with regard to the use of pharmacological factors, has been applied to the clinic for the treatment of β-hemoglobinopathies, especially for the homozygous Sickle Cell Disease (SCD) patients and double heterozygotes with β-thalassemia and SCD (β-thal/SCD). Hydroxyurea (HU) or hydroxycarbamide is the major therapeutic agent used for the management of these patients, approved by the Food and Drug Administration (FDA) in severely affected adults, since 1998. Of the key parameters of HU action in patients suffering from SCD and β-thal/SCD is the successful reproduction of HbF, which improves their pathological phenotype resulting in reduced or eliminated transfusion needs, though with controversial results, because of the moderate and heterogeneous HbF expression levels produced and the lack of specificity [9]. Recent evidence supports the suppressive role of LFR/*ZBTB7A* to the reinduction of HbF*,* among the non-responders group of SCD and β-thal/SCD patients to HU, suggested to depend on an epigenetic mechanism and mediated by the HU driven hypomethylation of its 5’ CpG island and the consequent elevated expression of LRF/*ZBTB7A*. Recognition binding sites for the LRF/*ZBTB7A* are apparent at CpG islands settled at the 5’ areas of several “modifier genes” of the β-globin genes’ cluster, such as the erythroid-specific transcription factors KLF1 and GATA2, the regulators of hematopoiesis and erythropoiesis MYB, SIN3A, and BCL11A, members of the MAPK signalling pathway (*MAP3K5*), and the *ZBTB7A* gene itself, implying an auto- and inter-regulatory role for this TF [2] within specific gene networks.

Furthermore, LRF/*ZBTB7A* has been demonstrated to directly bind the γ-globin genes’ promoter and known recognition sequences responsible for the Hereditary Persistence of Fetal Hemoglobin (HPFH) syndrome. HPFH is a benign condition which is characterized by high HbF expression throughout adulthood, and regarding SCD and β-thal/SCD patients, it plays a pivotal role in ameliorating the severity of their symptoms. HPFH-associated mutations ordinarily disrupt the LRF/*ZBTB7A* binding sites and thus raise the γ-globin gene expression, constituting an interesting therapeutic goal aiming to reactivate the developmentally silenced γ-globin gene expression [10].

### Lymphoid lineage fate decisions directed by LRF/*ZBTB7A*

LRF/*ZBTB7A* has been reported to regulate B versus T lymphocyte fate decisions by interfering with the Notch signaling pathway [11]. B cell development involves the sequential transition from the common lymphoid progenitors (CLP) to pro-B cell (progenitor B cell), pre-B cell (precursor B cell), immature naïve B cell, transitional naïve B cell, and ultimately mature B cell. The immature B cells normally migrate from the bone marrow to the secondary lymphoid organs in the body’s periphery, thereby reinforcing the ongoing maturation process [12]. During fetal lymphopoiesis in mice embryos, homozygous *Zbtb7a* deletion affects the early B cell development and leads to a significant reduction of the Pro-B stage cells, whereas HSC and CLP cell populations remain intact. However, in adult mice, the inducible inactivation of Lrf/*Zbtb7a* results in the drastic reduction of circulating B^220+^ cells and in the extrathymic double-positive (CD4/8) T cell development, limited in the bone marrow, at the expense of B cell development [13]. Furthermore, LRF/*ZBTB7A* prevents HSCs from premature differentiation towards T cell and supports adult HSC homeostasis by suppressing the Notch-mediated signals [14].

T cell differentiation process in the thymus is subcategorized into discrete stages based on the expression of the co-receptor molecules CD4 and CD8. The earliest thymocytes, which are CD4^-^/8^-^ negative (double negative, DN) differentiate into CD4^+^/8^+^ positive (double positive, DP) and further mature in CD4^+^ single positive expressing Thpok (T-helper inducing POZ/Krüppel-like factor) encoded by the *Zbtb7b* gene or in CD8^+^ single positive cells expressing the Runx3 TF [15]. Inactivation of both Thpok/*Zbtb7b* and Lrf/*Zbtb7a* in mice, revealed that Thpok is required for the intrathymic T regulatory (Treg) differentiation, and both TFs redundantly promote CD4^+^ T cell lineage maintenance [16] and Treg-mediated immune homeostasis. Thpok/*Zbtb7b* and Lrf/*Zbtb7a* deletion in Treg cells leads to a lethal inflammatory disease similar to that of Scurfy mice which carry a missense mutation in the Foxp3 gene and therefore lack functional CD4^+^Foxp3^+^ Treg cells. Thpok/*Zbtb7b* and Lrf/*Zbtb7a* support Foxp3-directed gene expression in Tregs, specifically through activation of IL-2-dependent genes [17, 18].

Activation of the conserved Notch signaling pathway is essential for the T cell differentiation, but is also critical for distinct cell fate decisions, in the secondary lymphoid organs, during the transition of the long-lived mature B cell pool towards the follicular B cells (FOB) versus the marginal zone B cells (MZB). Lrf opposes Notch function under normal conditions as defined in Lrf/*Zbtb7a* conditional knockout mice, which showed excessive MZB differentiation against FOB, though inactivation of the Delta-like 4 (DLL4), component of the Notch axis, rescued the aberrant lymphoid differentiation and the balance between MZB and FOB development was restored [19].

The concurrent elevated expression levels of LRF/*ZBTB7A* and BCL6 (*ZBTB27*), another member of the ZBTB family, have been reported to regulate the formation of Germinal Centers B (GCΒ) cells. GCs are formed by rapidly proliferating B cells after activation of naïve B cells through interaction with helper T cells and antigen-presenting cells and appear as a distinct histologic structure found in secondary lymphoid organs [20, 21]. Defected GC formation was obtained in conditional inactivated Lrf/*Zbtb7a* adult mice, which interestingly was not rescued by Notch loss, suggesting that Lrf is required for the maintenance and function of GCB cells rather than commitment to this stage. Downregulation of the tumor suppressor gene *p19*^*Arf*^ was confirmed to be responsible for the impaired proliferation and increased apoptosis seen in Lrf/*Zbtb7a*-deficient GCB-cells in mice [19].

Collectively, LRF/*ZBTB7A* plays a vital role to lineage cell fate decisions within the hematopoietic compartment [22, 23] illustrated in Fig. 1.

### LRF/*ZBTB7A* function in hematological cancers and solid tumors

#### Direct and indirect involvement of LRF/*ZBTB7A* in glycolysis

To meet the demands of a highly proliferative state and survival in various unfavorable microenvironments, tumors undergo fundamental alterations in their metabolism regarding carbohydrates, lipids, and glutamine [24] herein presenting, dependence on glycolytic ATP as the major energetic pathway and elevated de novo lipid synthesis to provide building blocks for membrane biosynthesis [25]. The most prominent aspect of malignant metabolic transformation is the glycolytic phenotype or the Warburg effect, whereby cancer cells exhibit high glycolytic activity under aerobic conditions [26, 27] and favor glycolysis, although it yields lower amounts of ATP than mitochondrial oxidative phosphorylation (OXPHOS) [28]. The pentose phosphate pathway (PPP) acts as an auxiliary secondary pathway to produce both NADPH and ribose-5-phosphate (R5P) for biosynthetic reactions and nucleic acids synthesis and it is enhanced by the increased aerobic glycolysis rate [28].

**Fig. 2:**
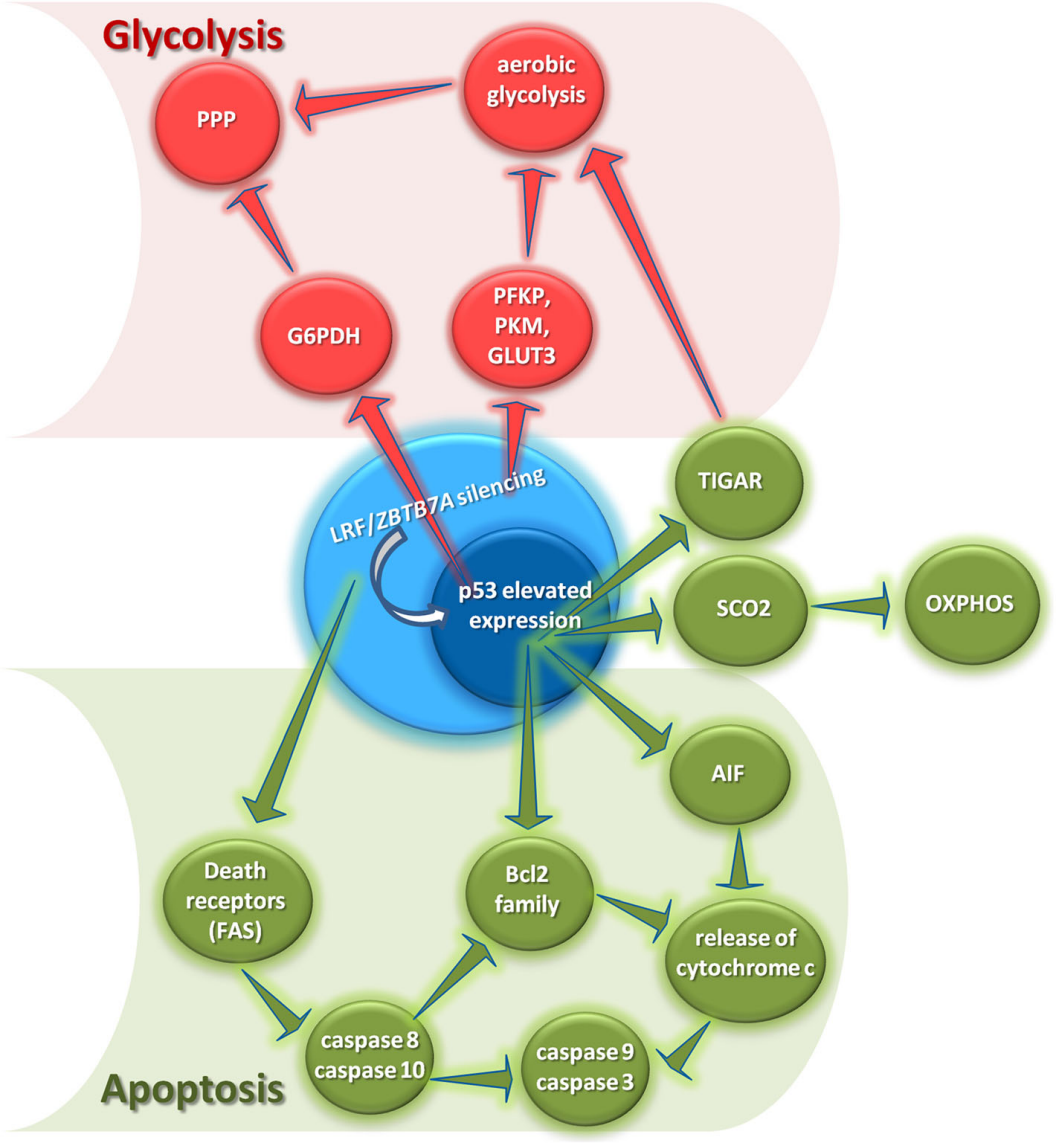
LRF/*ZBTB7A*’s silencing compromises Warburg effect and induces apoptosis in cancer cells. The inhibition of aerobic glycolysis and concomitant activation of oxidative phosphorylation (OXPHOS) is promoted by the LRF/*ZBTB7A*’s silencing and the subsequent activation of the expression and phosphorylation of p53, leading thus to compromised Warburg effect in cancer cells. Furthermore, LRF/*ZBTB7A*’s silencing induces apoptosis in cancer cell lines by mediating both known apoptotic pathways (intrinsic and extrinsic) as well as their cross-talk. That is, upon LRF/*ZBTB7A*’s silencing and the subsequent activation of p53, the pro-apoptotic Bcl-2 family proteins as well as AIF are activated and promote cytochrome c release from mitochondria and subsequent activation of caspase-9 and caspase-3 (intrinsic apoptotic pathway). Meanwhile, LRF/*ZBTB7A*’s silencing promotes the expression of the Fas receptor (death receptor), leading to the activation of the downstream caspase-10 and caspase-8 and leading thus to activation of the extrinsic apoptotic pathway. In addition, caspace-8 further enhances the activation of Bcl2 family members, as well as the activation of caspase-9 and caspase-3 supporting the hypothesis that LRF/*ZBTB7A*, besides affecting both apoptotic pathways, potentially mediates the cross-talk between the intrinsic and extrinsic apoptotic pathways in cancer cells (in red color are cellular processes inhibited and in green color are cellular processes activated by LRF/*ZBTB7A* silencing). *A*bbreviations: AIF, apoptosis inducing factor; FasR, Fas receptor; G6PDH, glucose-6-phosphate dehydrogenase; OXPHOS, oxidative phosphorylation; PPP, pentose phosphate pathway; SCO2, cytochrome c oxidase 2; TIGAR, TP53-induced glycolysis and apoptosis regulator

Current advances in molecular biology and cancer genetics provide evidence for mechanistic links between dysfunction of oncogenic proteins or tumor suppressors and hyperactive glycolysis in cancer [29]. *KRAS* and *MYC* proto-oncogenes, protein kinase B (*AKT)*, epidermal growth factor receptor (*EGFR*), BCR-ABL fusion gene, and the receptor tyrosine kinase (*ALK*) promote independently glycolysis via upregulation of various glycolytic enzymes [30–32] or intermediates including glucose transporters I and III (*GLUT1, GLUT3*) [33, 34]. In addition, many oncoproteins activate the hypoxia inducible factor (HIF) via hypoxia-independent mechanisms or a pseudohypoxic state to enhance tumor glycolysis [30], while several glycolytic enzymes, including hexokinase 2 (*HK2*), phosphofructokinase 2 (*PFK2*), pyruvate kinase M2 (*PKM2*), lactate dehydrogenase A (*LDHA*), and pyruvate dehydrogenase kinase (*PDK*), have been identified as HIF-targeted genes [35]. Contrariwise, the tumor suppressor p53 promotes mitochondrial respiration through multiple mechanisms and ultimately dampens aerobic glycolysis mainly by inducing the cytochrome c oxidase (SCO2) and the *TP53*-induced glycolysis and apoptosis regulator (TIGAR), respectively [36]. The LRF/*ZBTB7A’s* silencing has been shown to induce p53 expression and phosphorylation [37, 38] indicating that LRF/*ZBTB7A* loss-of-function indirectly inhibits aerobic glycolysis and enhances OXPHOS, compromising thus the Warburg effect in cancer cells. However, Liu et al. [34] demonstrated that LRF/*ZBTB7A* mediates the transcriptional repression of glycolytic genes, including *GLUT3*, phosphofructokinase *(PFKP)*, and *PKM*, indicating a second, opposing to the first, direct nuclear upregulating effect of LRF/*ZBTB7A’s* silencing on glycolysis. Whether and which mechanism predominates in the cellular transformation extended in a tissue- or cell-specific phenomenon, remains to be elucidated. Additionally, it has been shown that p53 inhibits the diversion of glycolytic intermediates into the PPP by binding and inhibiting glucose-6-phosphate dehydrogenase (*G6PDH*) [36]. To this end, LRF/*ZBTB7A’s* silencing is expected to further compromise PPP indirectly via p53-dependent inhibition of G6PDH (Fig. 2).

#### LRF/*ZBTB7A* as an oncogene

LRF/*ZBTB7A* has been characterized as an oncogene in many human cancers. It has been shown to be overexpressed in most of the human non-Hodgkin lymphoma cases (NHL) and commonly in conjunction with BCL6 (*ZBTB27*) [38]. Furthermore, frequent mutations of the LRF/*ZBTB7A* zinc finger coding sequences are identified in acute myeloid leukemia (AML) with translocation t(8;21), which links to a co-operational mechanism of action with the *RUNX1/RUNX1T1* fusion gene towards leukemogenesis [39].

However, LRF/*ZBTB7A* association with cancer progression goes far beyond hematological cancers. After the initial report associating LRF/*ZBTB7A* with T cell lymphoblastic lymphoma/leukemia through the suppression of the *Arf* tumor suppressor (*p19*^*Arf*^) gene and the consequent decrease of p53 activity [38], accumulating evidence further show that LRF/*ZBTB7A* is overexpressed in several other human cancers, including non-small cell lung cancer (NSCLC) [40–43], hepatocellular carcinoma [44–48], prostate [49, 50], ovarian [51], breast [52–54] and gastric cancers [55], glioma [56], sarcomas [57, 58], colorectal cancer [59–62], and renal carcinoma [63]. Further elucidation of these oncogenic functions revealed that LRF/*ZBTB7A* can influence cancer cell survival and proliferation, apoptosis, invasion and migration/metastasis, traits comprising some of the key biological capabilities required for the multistep development of human cancer, also presented as “hallmarks of cancer” [64]. LRF/*ZBTB7A* expression also positively correlates with many clinicopathologic parameters of human cancers like tumor size, histological grade, and overall patient survival, findings rendering this molecule a potential biomarker for human cancers, as well as an attractive therapeutic target.

In order to exert these functions, LRF/*ZBTB7A* interacts with or regulates many different binding partners, forming a complex network of downstream pathways. These data highlighted not only the diversity of its roles but also their context-dependency. Cell cycle-related genes are frequent targets of LRF/*ZBTB7A* [48, 56, 57, 59, 65–67], offering a mechanistic explanation of the factor’s ability to affect cell proliferation. In human hepatocellular carcinoma cell lines, LRF/*ZBTB7A* regulates cell cycle progression by either suppressing or promoting the expression of key cell cycle regulators like cyclin-dependent kinase inhibitors 2B, 1B, and 1A (*p15, p27,* and *p21* genes, respectively), tumor protein p53 (*TP53*), CYCLIN D1, CYCLIN D3, and cyclin-dependent kinases CDK4 and CDK6. Commonly, LRF/*ZBTB7A* has been shown to promote progression through the S phase of the cell cycle in colorectal, osteosarcoma, and cervical cancer cells by transcriptionally repressing *p21* [59, 68], whereas its depletion induces G1 cell cycle arrest through increased expression of *p53* and *p21* in chondrosarcoma and lung cancer cells [57, 59, 69]. Additional cell cycle-related transcriptional targets of LRF/*ZBTB7A* include the well-known tumor suppressor retinoblastoma (*RB*) gene, and by negatively regulating its transcription, LRF/*ZBTB7A* can alleviate the cell-cycle arrest effect of RB [70].

Suggested underlying mechanisms for these functions, at least in hepatocellular carcinoma, involve the phosphoinositide-3-kinase-protein kinase B/Akt (PI3K/Akt) and RAS/RAF/MEK/ERK pathways [65, 66]. The PI3K/Akt pathway is activated in response to extracellular stimuli through the high-affinity cell surface receptor tyrosine kinases and has multiple downstream targets regulating among others cell growth and survival, cell cycle progression and proliferation, apoptosis, and glycogen synthesis [71, 72]. Along this pathway, LRF/*ZBTB7A* was found to inhibit phosphatase and tensin homolog (PTEN), one of its negative regulators, thereby enabling an incessant signaling within cells with aberrant consequences [66].

The RAS/RAF/MEK/ERK pathway involves a cascade of events starting with an extracellular mitogen and usually resulting in the regulation of cell proliferation and division, through the consecutive activation of proto-oncogenes *KRAS* and *c-RAF* (GTP-ase and serine/threonine kinase respectively), mitogen-activated protein kinases, and extracellular signal-regulated kinases, also known as mitogen-activated protein kinases (*MAPK*), which can eventually activate the expression of transcription factors responsible for cell proliferation. Accumulating evidence supports the idea that blockade of one pathway enhances the other via key components participating in both pathways and implying potential cross-talk mechanisms between them [71, 73]. As both pathways are deregulated in many human cancers [71], the key participants of these signaling cascades, including LRF/*ZBTB7A*, represent attractive targets for therapeutic interventions.

Closely related to the effects on cell cycle progression are the LRF/*ZBTB7A’s* implications in regulating senescence and apoptosis [37, 57, 74]. LRF/*ZBTB7A* deletion can urge cells to attain senescence through upregulation of *p21* and *p53* [57]. *Zbtb7a*-knockout mouse embryonic fibroblasts also exhibit premature growth arrest and subsequent senescence caused by upregulated *p19*^*Arf*^ and *Trp53* [38]. On the other hand, overexpression of LRF/*ZBTB7A* can rescue this phenotype and maintain cells in a proliferative state, by enhancing E2F-dependent transcription and upregulation of *CYCLIN E* [74].

In hepatocellullar carcinoma cells, silencing of LRF/*ZBTB7A* increased p53 expression and initiated caspase-dependent apoptosis via death receptor- and mitochondria-mediated pathways [37], whereas in breast cancer, LRF/*ZBTB7A* anti-apoptotic function involved Survivin, a negative regulator of apoptosis [52], discussed in a separate section of this review.

LRF/*ZBTB7A* overexpression is also associated with enhanced cancer cell invasiveness and metastasis. Potential mechanisms involve the LRF/*ZBTB7A*-mediated transcriptional activation of myocyte enhancer factor 2D (*MEF2D*), an oncogene favoring the invasion of hepatocellular carcinoma cells [46, 47], and independently, the activation in ovarian cancer of membrane type 1-matrix metalloproteinase (*MT1-MMP*), a molecule playing a vital role in the dissociation of the extracellular matrix and the consequent dissemination of tumor cells [51].

Other targets of LRF/*ZBTB7A*-mediated regulation of oncogenesis include Striatin 4 (*STRN4*), a protein implicated in the progression of various human cancers [50, 75] and protein C-ets-1 (*ETS-1*), which is overexpressed in several cancers and implicated in cell proliferation, apoptosis, invasion, and migration [61]. Additionally, in breast cancer cells, LRF/*ZBTB7A* has been proposed to participate in transforming growth factor beta (TGF-β) signaling, a critical pathway for tumor progression [76], by binding to specificity protein 1 (SP1) and downregulating Smad4 expression [77]. A repressive effect on the TGF-β pathway was also attributed to the recruitment of HDAC to the Smad4 complex mediated by the POZ domain of LRF/*ZBTB7A* [78]. LRF/*ZBTB7A* has also been described as a target of key tumorigenic cascades like epidermal growth factor (*EGF*), SP1, endothelial PAS domain-containing protein 1 (*EPAS-1*), and TGF-β pathways [79–82]. Furthermore, LRF/*ZBTB7A* seems to be a frequent target of various microRNAs (miRNAs) during carcinogenesis, as miR21, miR100, miR125, miR137, miR520e, and miR663 are indicated as modulators of the LRF/*ZBTB7A* expression levels in many different cancers [44, 46, 55, 58, 63, 69, 83–85]. Finally, gene amplification was suggested as a potential mechanism driving overexpression of LRF/*ZBTB7A* in non-small-cell lung carcinoma, accompanied by transcriptional and post-translational aberrations [43].

It is therefore plausible that, at least in some human cancers, LRF/*ZBTB7A* represents one of the incipient traits rendering cancer cells tumorigenic and ultimately malignant, thus favoring cancer progression.

#### LRF/*ZBTB7A* as a tumor suppressor

LRF/*ZBTB7A* plays an even more multifaceted role in carcinogenesis. A number of studies have indicated that LRF/*ZBTB7A* exerts also tissue- and context-dependent oncosuppressive functions. LRF/*ZBTB7A’s* expression was found to be repressed by the heterochromatin protein 1γ (HP1γ), which localizes to both the heterochromatic and euchromatic regions within the cell nucleus and is known to be involved in gene expression regulation [86]. Supporting evidence shows that LRF/*ZBTB7A’s* depletion restores the proliferation and migration defects caused by HP1γ upregulation in lung adenocarcinoma cells, linked to a poor prognosis in patients. Furthermore, HP1γ-induced downregulation of the LRF/*ZBTB7A* favors the expression of several tumor-promoting factors, such as AXL receptor tyrosine kinase (*AXL*), plasmacytoma variant translocation 1 (*PVT1*), and ETS Like-1 protein (*ELK1*) [87].

Lrf/*Zbtb7a* was also shown to interact with and antagonize the transcriptional activity of Sry-related HMG box 9 (*Sox9*), which drives expression of several genes contributing to increased proliferation and oncogenesis, and that loss of Lrf/*Zbtb7a* bypasses cellular senescence and promotes invasion in *Pten*-null prostate cancers [88, 89]. Interestingly, this finding was shown to have clinical implications, as analysis of human prostate cancer samples showed that LRF/*ZBTB7A* expression can stratify patients’ response to androgen-deprivation therapy [22, 90, 91]. Through *SOX9*, LRF/*ZBTB7A* also affects the commitment of adult mesenchymal stem cells towards undifferentiated sarcomas [92], whereas in breast cancer, LRF/*ZBTB7A* regulates estrogen receptor’s alpha (ERα) expression, one of the major markers used to determine course of treatment, and predicts a favorable/unfavorable outcome for patients treated with endocrine therapies [93].

In vitro and in vivo studies with hepatocellular carcinoma cells revealed another regulatory pathway of LRF/*ZBTB7A* expression, mediated by miR106, and showed that LRF/*ZBTB7A* overexpression, caused by miR106 inhibition, abrogates cell growth [83]. Further oncosuppressive functions of LRF/ZBTB7A include S cell cycle arrest, promotion of apoptosis and repression of migration in gastric cancer [94], and suppression of metastasis through transcriptional repression of melanoma cell adhesion molecule (MCAM) in melanoma [95]. An additional interesting mechanism related to the LRF/*ZBTB7A’s* oncosuppressive functions involves the transcriptional repression of key oncogenic glycolytic genes like glucose transporters 1 and 3 (*GLUT1, GLUT3*), phosphofructokinase (*PFKP*), and pyruvate kinase muscle isozyme (*PKM*), thereby inhibiting cancer metabolism [33, 34].

#### Cooperation of LRF/*ZBTB7A* with NF-κB

Another significant interaction mediating LRF/*ZBTB7A* functions in cancer is through binding and promoting the signaling of nuclear factor (NF)-κB, a transcription factor with well-known properties in regulating many aspects of cancer, inflammation, and immune responses [96]. NF-κB signaling can be activated in both immune and non-immune tissues by various extracellular signals and positively or negatively modulate the cell proliferation and apoptosis [97]. NF-κB signaling comprises a canonical pathway, involving the subunits p50/p65, the activating kinase IKKβ and the inhibitor IκBα, and a non-canonical pathway, involving the subunits RelB/p52, the activating kinase IKKα and the inhibitor IκBβ. Activation of NF­κB is transiently induced in response to various stressful stimuli like infections, DNA damage or pro-inflammatory cytokines, and pathogen- and damage-associated molecular pattern (PAMPs and DAMPs) [96]; however, in cancerous tissues, NF-κB signaling can be constitutively active [96]. LRF/*ZBTB7A* was found to impact the transcription of p65 and IκBα [3, 98], i.e., members of the canonical pathway of NF-κB signaling and has been proposed to positively affect transcription of NF-κB target genes by increasing nuclear localization of the p65 subunit of NF-κB [99], as well as by facilitating the accession of NF-κB to its target genes [3].

#### Additional pathways of LRF/*ZBTB7A* functions, implicated in cancer

Interestingly, accumulating evidence reveals a fascinating plurality of LRF/*ZBTB7A* roles, in a transcription-independent manner of function. In a recent study, LRF/*ZBTB7A* was described to hold a key role in maintaining genome integrity by regulating the non-homologous end joining pathway of double-strand break DNA repair [100]. Along the same line, LRF/*ZBTB7A* was also found to participate in alternative splicing modulation [101]. Another unexpected function of the *ZBTB7A* gene was recently reported, showing that the locus can actually produce two different types of RNA, a linear one and a non-coding circular one, which can nonetheless contribute to cancer progression independently from and possibly antagonistically to the linear counterpart, further reinforcing LRF/*ZBTB7A* tumorigenic implications [102]. Strikingly, LRF/*ZBTB7A* was also found to define the tumor microenvironment and the influx of tumor infiltrating immune cells in prostate cancer, and in particular the attraction of polymorphonuclear cells [103]. Finally, the chromosomal region 19p13.3, encompassing the LRF/*ZBTB7A,* is frequently lost in human cancers, as presented in the Cancer Genome Atlas database (PMID: 22901813), while somatic loss-of-function zinc finger mutations of LRF/*ZBTB7A* present another mechanism promoting cancer progression [104].

Collectively, the data presented so far suggest that LRF/*ZBTB7A* is a pivotal factor regulating many different aspects of cancer progression (Fig. 2 and Table 1).

**Table 1:** Summary of LRF/*ZBTB7A* functions in cancer. The protein is notorious for exerting tissue- and context-dependent oncogenic or oncosuppressive functions, through multiple molecular interactions

Cancer	Function	Molecular partners	Biological process	Reference
Lymphoma	Oncogenic	BCL6‚ p19^Arf^	Cell transformation, cell growth	38
Acute myeloid leukemia	Oncogenic	RUNX1/RUNX1T1	Proliferation	39
Lung cancer	Oncogenic	miR-125a, miR-520e	Cell cycle, cell growth, proliferation, apoptosis, invasion	40-43, 69, 85
	Oncosuppressive	HP1γ, AXL, PVT1, ELK1	Proliferation, migration	88
Hepatocellular carcinoma	Oncogenic	miR-125, miR-137, MEF2D, p53, p21,p27, cyclins, CDKs, PI3K/AKT, RAS/RAF/MEK/ERK, BCL2, FAS receptor, FADD, caspase-8, caspase-10	Cell cycle, proliferation, apoptosis, migration/metastasis, invasion	37, 44-48, 65, 66
	Oncosuppressive	miR-106b	Proliferation, apoptosis	83
Prostate cancer	Oncogenic	STRN4, EGF	Proliferation, apoptosis	49, 50, 79
	Oncosuppressive	Sox9, AR	Senescence, proliferation, invasion	89, 92
Ovarian cancer	Oncogenic	MT1-MMP	Migration/metastasis, invasion	51
Breast cancer	Oncogenic	Survivin, SP1,Smad4	Proliferation, apoptosis	52-55, 77
Gastric cancer	Oncogenic	C/EBPα/ miR-100	Proliferation, migration/metastasis	55
	Oncosuppressive	Not defined	Cell cycle, apoptosis, migration/metastasis	95
Glioma	Oncogenic	AKT, cyclins, CDKs, NF-κB, Survivin	Cell cycle, proliferation, apoptosis, migration/metastasis	56
Sarcoma/mesenchymal tumors	Oncogenic	p21, p53, miR-663a, LncRNA GAS5, circular RNA of LRF/*ZBTB7A* - ILF2/3	Cell cycle, proliferation, apoptosis, migration/metastasis, invasion, angiogenesis	57, 58, 68, 103
	Oncosuppressive	DLK1, Sox9	Cell transformation, proliferation	93, 103
Colorectal cancer	Oncogenic	DAP5/p53, ETS-1	Cell cycle, proliferation, apoptosis, migration/metastasis, invasion	59-62
Renal carcinoma	Oncogenic	miR-137	Proliferation, invasion	63
Liver cancer	Oncogenic	miR-21, Sprouty1	Cell growth, proliferation	84
Bladder cancer	Oncogenic	TGF-β	Cell growth, migration/metastasis, invasion	81
Melanoma	Oncosuppressive	MCAM	Migration/metastasis	96

#### LRF/*ZBTB7A* silencing facilitates apoptosis

Recent studies show that LRF/*ZBTB7A* is capable of promoting apoptosis via the p53 pathway [37, 68]. To this end, LRF/*ZBTB7A* acts as a master administrator of cellular transformation and proliferation and its silencing potently induces the p53 pathway and the two subsequent apoptotic signaling pathways: (1) the mitochondria-mediated (intrinsic) and (2) the death receptor-mediated (extrinsic) pathway, thought to be distinct until recently [37].

The intrinsic, mitochondrial apoptotic pathway is regulated by the Bcl-2 family of proteins that administrate the release of cytochrome c from the mitochondria [105]. Bcl-2 pro-apoptotic (Bax, Bak, Bad, Bid, Bik, and Bim) protein family promotes the release of cytochrome c from the mitochondria, which initiates the apoptotic cascade by activating caspase-9, followed by the cleavage and activation of downstream effector proteases, such as caspase-3 [106]. Once activated, caspase-3 cleaves the Poly (ADP-ribose) polymerase-1 (PARP-1) into p89 and p24 fragments, promoting DNA fragmentation and eventually triggering cell apoptosis [107]. Elevated expression of p53, pro-apoptotic Bcl-2 family proteins and corresponding changes in other apoptosis-related factors, including apoptosis inducing factor (*AIF*) expression levels and cytochrome c release from mitochondria, other apoptosis-related factors derived from the *ZBTB7A*-knockdown HepG2 cell line support the proposed intrinsic mechanism [37]. Besides, the extrinsic apoptotic pathway is mediated by death receptors. Fas ligand interacts with the Fas receptor, causing caspase-8 and caspase-10 activation, which subsequently cleave directly and activate downstream effector proteases, such as caspase-3, causing cell apoptosis [108–111]. Upon LRF/*ZBTB7A*’s silencing the expression of the Fas receptor is increased, leading to the activation of the downstream caspase-10 and caspase-8, as were evidently upregulated in the *ZBTB7A*-knockdown HepG2 cell line compared to the controls [37].

In addition, caspase-8 and caspase–10 may cleave the Bcl-2 family member Bid into truncated Bid (tBid) and thus resulting in disruption and release of cytochrome c [112, 113] (Fig. 2). It is therefore speculated that LRF/*ZBTB7A* potentially mediates the cross-communication between the intrinsic and extrinsic apoptotic pathways, notably in cancer cells.

### LRF/*ZBTB7A* expression levels during adipogenesis

Ιn vitro differentiation of preadipocytes is a step-forward process. That is, upon reaching confluence, preadipocytes become contact-inhibited and proliferation stops at the G1/S phase boundary. Following hormonal induction, preadipocytes complete two cycles of cell division known as mitotic clonal expansion (days 1–2), and finally, after a second growth arrest (days 3–4), they undergo terminal differentiation (days 4–10) resulting in the expression of genes defining the adipocyte phenotype [114]. The molecular mechanisms regulating the transition between cellular proliferation and differentiation of preadipocytes remain in part elusive.

**Fig. 3:**
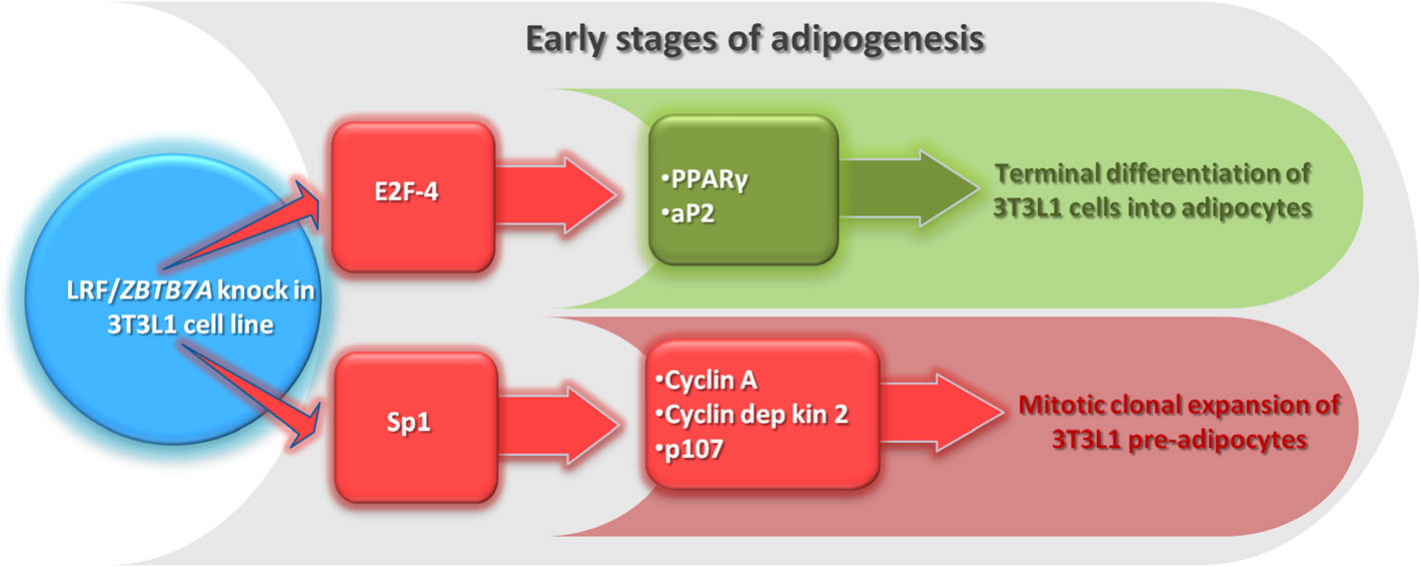
LRF/*ZBTB7A* switches 3T3-L1 preadipocytes’ fate from cellular proliferation to terminal differentiation. Human LRF/*ZBTB7A* expression in 3T3-L1 preadipocytes promotes cell growth arrest at the stage of mitotic clonal expansion via inhibition of Sp1-dependent activation of cyclin A, cyclin-dependent kinase 2, and p107, while indirectly (via E2F-4 inhibition] activates PPARγ and aP2 transcription factors, known to be involved in terminal differentiation of 3T3-L1 cells into adipocytes (in red color are cellular processes inhibited and in green color are cellular processes activated by LRF/*ZBTB7A* expression). *A*bbreviations: aP2, adipocyte fatty acid-binding protein; E2F-4, E2F transcription factor 4; PPARγ, peroxisome proliferator-activated receptor gamma; Sp1, specificity protein 1

Ιn human adipose tissue, LRF/*ZBTB7A* mRNA is normally expressed, though the highest levels are found in samples from morbidly obese subjects as displayed in oligonucleotide microarray experiments, originally designed to identify novel rosiglitazone [oral antidiabetic drug that activates gamma isoform of peroxisome proliferator-activated receptor (PPARγ)] targeted genes in early human preadipocyte differentiation [115, 116]. Consistently, in similar experiments, using murine 3T3-L1 preadipocytes over-expressing Lrf/*Zbtb7a*, it was evident that PPARγ and aP2 mRNAs were both significantly upregulated [117], and this induction was related to the early phase of differentiation process (days 2-4), urging terminal cell differentiation towards adipogenesis. Furthermore, murine cell lines constitutively expressing Lrf/*Zbtb7a* showed evidence for accelerated adipogenesis with earlier induction of differentiation markers and enhanced lipid accumulation, suggesting that this TF contributes significantly in the differentiation process due to downregulation of E2F-4 [117], a transcriptional factor known to inhibit PPARγ expression [118]. Since the LRF/*ZBTB7A* protein levels peak at the end of mitotic clonal expansion, it has been hypothesized that this TF facilitates the cells’ terminal differentiation during adipogenesis. Indeed, 3T3-L1 cells stably overexpressing Lrf/*Zbtb7a* showed reduced DNA synthesis and reduced expression of cyclin A, cyclin-dependent kinase 2, and p107, proteins known to be involved in the regulation of mitotic clonal expansion. In addition, Lrf/*Zbtb7a* reduced the transcriptional activity of the cyclin A promoter [117]. Similarly, human LRF/*ZBTB7A* reduces the activity of cyclin A promoter indirectly by inhibiting Sp1 while represses the activity of E2F-4 promoter directly [119] (Fig. 3). Taken together, LRF/*ZBTB7A* is induced during the early phases of human and murine preadipocyte differentiation where it contributes to adipogenesis through influencing the switch from cellular proliferation to terminal differentiation by exhibiting thus a dual function of promoting both growth arrest and terminal adipocyte differentiation.

### ZBTB7A and insulin: a futile cycle

Type 1 diabetes (T1D) is a complex disease with a strong genetic background, and there are more than 40 loci identified to correlate with increased T1D susceptibility [120, 121]. Interestingly, insulin self-tolerance is, to a large extent, assured by the expression of small quantities of insulin by medullary thymic epithelial cells (mTECs) [122, 123]. The transcriptional regulation of thymic insulin gene expression was poorly understood until recently, where it has been confirmed the binding of specific TFs, namely LRF/*ZBTB7A*, AP-1 (*JUN*), and EWS RNA binding protein 1 (*EWSR1*), to the human insulin gene promoter alone or joined to a class I or class III VNTR allele, located 596 bp upstream of the insulin gene [124–126]. The shortest (class I) VNTR allele, consisting of 26 to 63 repeats, predisposes to T1D, while the long (class III) VNTR allele, consisting of 140 to 210 repeats, reduces 2- to 4-fold the risk for T1D compared to class I/I homozygotes [127, 128]. The selection for self-tolerant T cells takes place in the thymus and requires the AIRE, the autoimmune regulator, responsible for the expression of thousands of tissue-restricted antigens (TRAs) in a specialized subset of medullary thymic epithelial cells (mTECs) [129]. The AIRE-dependence of thymic insulin expression has been directly demonstrated in the mouse [130, 131] and indirectly in the human [132].

All three proteins (LRF/*ZBTB7A*, AP-1, and *EWSR1*) could induce insulin expression in transfected HEK-293 cells, but LRF/*ZBTB7A* provided the most robust results especially in the presence of the AIRE, with an additional 11-fold increase of the insulin mRNA levels from a co-transfected reporter driven by the class III VNTR allele. Consequently, LRF/*ZBTB7A* was identified as a strong candidate for the regulation of thymic insulin expression [133]. On the other hand, it has been shown that insulin stimulates the murine *Zbtb7A* promoter activity and enhances endogenous *Zbtb7A* mRNA and protein levels in a dose- and time-dependent manner. Furthermore, the PI3K/AKT cascade and transcription factor Sp1, known to recognize and bind methylated regions in genes’ promoter(s) [134], have been implicated to the observed insulin-induced Lrf/*Zbtb7a* expression in HepG2 cells [135].

## Conclusions

LRF/*ZBTB7A* presents pleiotropic actions and is involved in the regulation of many fundamental physiological cell processes as follows: the terminal erythrocyte and adipocyte differentiation, the lineage cell fate decisions of pre-mature and mature B and T cells, the insulin self-tolerance, and also, pathophysiological conditions such as numerous cancer types.

Collectively, LRF/*ZBTB7A* is rising to a central player controlling proper cell proliferation and differentiation by participating in several molecular mechanisms and interacting with an impressive variety of binding partners in a tissue- and context-dependent manner to exert either oncogenic or oncosuppressive functions. Altered expression profile of LRF/*ZBTB7A* has been detected in several types of cancer, supporting the idea that it potentially bridges discrete downstream cell signaling pathways as well as intrinsic/extrinsic apoptotic pathways via regulation of mutual participants. To this end, LRF/*ZBTB7A* functions mainly as a co-operator with other TFs rather than a master or “pioneering” TF and does not directly activate or repress gene promoters as has been established for NF-κB or the Hypoxia Inducible Factor (*HIF*), but alternatively influences the accessibility of other TFs and DNA-binding proteins.

A critical LRF/*ZBTB7A* feature is its penchant for binding CG reach DNA sequences, located within regulatory gene regions and its evident ability to from homo- or hetero-dimmers capable to recruit chromatin remodeling complexes such as HDACs and NuRD. It remains to be clarified whether LRF/*ZBTB7A* belongs to the recently introduced generation of TFs, with the capability to sense epigenetic events and orchestrate reciprocal action as a response, which further transduces chromatin context and triggers changes in gene expression.

## Data Availability

Data sharing not applicable to this article as no datasets were generated or analyzed.

## Abbreviations

AKT: Akt serine/threonine kinase
AR: Androgen receptor
AXL: AXL receptor tyrosine kinase
BCL2: B-cell lymphoma 2
BCL6: B-cell lymphoma 6
C/EBPα: CCAAT/enhancer-binding protein alpha
CDKs: Cyclin-dependent kinases
DAP5: Death-associated protein 5
DLK1: Delta like non-canonical Notch ligand 1
EGF: Epidermal growth factor
ELK1: ETS Like-1 protein
ERK: Extracellular signal-regulated kinase
ETS-1: Protein C-ets-1
FADD: Fas-associated via death domain
FAS receptor: Fas cell surface death receptor
HP1γ: Heterochromatin protein 1γ
ILF2/3: Interleukin enhancer binding factors 2/3
LncRNA GAS5: Long non-coding RNA Growth arrest-specific transcript 5
MCAM: Melanoma cell adhesion molecule
MEF2D: Myocyte enhancer factor 2D
MEK: Mitogen-activated protein kinases
miR-100: microRNA-100
miR-106b: microRNA-106b
miR-125: microRNA-125
miR-125a: microRNA-125a
miR-137: microRNA-137
miR-137: microRNA-137
miR-21: microRNA-21
miR-520e: microRNA-520e
miR-663a: microRNA-663a
MT1-MMP: Membrane type 1-matrix metalloproteinase
NF-κB: Nuclear factor-κB
p19^Arf^: Cyclin-dependent kinase inhibitor 2A
p21: Cyclin-dependent kinase inhibitor 1A
p27: Cyclin-dependent kinase inhibitor 1B
p53: Tumor protein p53
PI3K: Phosphoinositide-3-kinase-protein kinase
PVT1: Plasmacytoma variant translocation 1
RAF: Raf-1 proto-oncogene, serine/threonine kinase
RAS: RAS oncogene family
RUNX1/RUNX1T1: RUNX family transcription factor 1/RUNX1 partner transcriptional co-repressor 1
Smad4: SMAD family member 4
Sox9: Sry-related HMG box 9
SP1: Specificity protein 1
STRN4: Striatin-4
TGF-β: Transforming growth factor beta
